# A New Hypoxia-Related Prognostic Risk Score (HPRS) Model Was Developed to Indicate Prognosis and Response to Immunotherapy for Lung Adenocarcinoma

**DOI:** 10.1155/2022/6373226

**Published:** 2022-07-30

**Authors:** Xiahui Yang, Minchao Liang, Zhiqi Yu, Jiaquan Fan

**Affiliations:** ^1^Department of Respiratory and Critical Care Medicine, The Second Affiliated Hospital of Guangzhou Medical University, PCODE:440100, Guangzhou, China; ^2^Department of Oncology, HaploX Biotechnology, 8 / F, Aotexin Power Building, No.1, Songpingshan Road, High Tech North District, Nanshan District, Shenzhen 110000, Guangdong Province, China

## Abstract

**Background:**

Hypoxia is a typical microenvironmental feature of most solid tumors, affecting a variety of physiological processes. We developed a hypoxia-related prognostic risk score (HPRS) model to reveal tumor microenvironment (TME) and predict prognosis of lung adenocarcinoma (LUAD).

**Methods:**

LUAD sample expression data were from The Cancer Genome Atlas (TCGA) and Gene Expression Omnibus (GEO) databases. Weighted gene co-expression network analysis (WGCNA) and least absolute shrinkage and selection operator (LASSO) Cox regression identified hypoxia-related genes (HRGs) to create HPRS. The prognostic value, genetic mutation and TME, and therapeutic response of distinct HPRS groups were analyzed. Univariate and multivariate Cox regression analysis identified independent factors associated with the prognosis of LUAD. A decision tree based on HPRS and clinicopathological variables was established using the classification system based on decision tree algorithm. A nomogram was constructed with important clinical features and HPRS by the RMS package.

**Results:**

A HPRS model with five HRGs was developed and verified in two separate cohorts of GEO. HPRS model divided patients with LUAD into two groups. High HPRS was related to high probability of genetic alterations. HPRS could predict the prognosis, TME, and sensitivity to immunotherapy/chemotherapy of LUAD. The decision tree defined four risk subgroups with significant OS differences. Nomogram with integrated HPRS and clinical features had acceptable accuracy in predicting LUAD prognosis.

**Conclusions:**

A HPRS model was developed to evaluate prognosis, genetic alterations, TME, and response to immunotherapy, which may provide theoretical reference for the study of molecular mechanism of hypoxia in LUAD.

## 1. Introduction

Lung cancer is the most frequently occurring cancer and the leading cause of cancer death in men, and the morbidity and mortality are approximately twice that of women [1]. 80% of lung cancers belong to nonsmall-cell lung cancer (NSCLC), which can be subdivided into adenocarcinoma, squamous cell carcinoma, bronchiolar alveolar carcinoma, and large cell carcinoma [2]. About 2/3 of lung cancer deaths worldwide can be attributed to smoking [1]. Among the main histological types of lung cancer, lung adenocarcinoma (LUAD) has the weakest correlation with smoking, which often occurs in women and nonsmokers [3]. However, most patients have advanced diseases at the time of diagnosis. At present, the main treatment methods are surgery, chemotherapy, and radiotherapy [4]. Although great progress has been made in oncology management of advanced lung cancer in recent years, the survival rate is still very low [5].

Oxygen is essential for energy metabolism to drive cellular bioenergetics. In the development of cancer, the supply of oxygen is limited by the rapid and uncontrolled proliferation of tumors. Therefore, oxygen deficiency is the most typical microenvironmental feature of almost all solid tumors [6]. Hypoxia has different effects on the progression of cancer. First of all, hypoxia is associated with tumor recurrence and chemotherapy resistance. Previous studies have revealed the molecular mechanism of hypoxia inducing drug resistance in colorectal cancer through HIF-1 *α*/miR-338-5p/IL-6 feedback loop [7]. Hypoxia induces resistance of lung cancer cells to EGFR inhibitors by upregulating FGFR1 and MAPK pathways [8]. A clinical study of hypoxia in prostate cancer shows that hypoxia is associated with early biochemical recurrence after radiotherapy and local recurrence of the prostate [9]. And hypoxia is associated with the biological progression of many cancers. Hypoxia-induced HIF-1 *α* and lncRNA-CF129 feedback promote proliferation and invasion of pancreatic cancer by stabilizing p53 protein [10]. Hypoxia induces ZEB1 to activate CCL8 transcription, which attracts macrophages through CCR2-NF-*κ*B pathway to promote the progression of cervical cancer [11]. Hypoxia-induced FOXO4/LDHA axis regulates glycolysis of gastric cancer cells [12]. Hypoxia-induced acetylation of PAK1 promotes autophagy and contributes to brain tumorigenesis via phosphorylating ATG5 [13]. Therefore, a comprehensive understanding of the biological effects of hypoxia-related regulatory molecules will be helpful to the treatment of cancer.

Tumor hypoxia also has been found to be a characteristic feature in lung cancer [14]. Lungs are directly exposed to higher oxygen concentrations than most other tissues. The oxygen concentration in normal lung tissue is about 5.6% O_2_, while the oxygen concentration in NSCLC is 1.9–2.2% [15]. Hypoxia supports carcinogenesis and tumor progression in two NSCLC histological subtypes involving different molecular pathways, resulting in hypoxic adaptation differences between LUAD and lung squamous cell carcinoma (LUSC) [16]. LUAD seems to be more often associated with hypoxic regions, such as hypoxic regions of lung adenocarcinoma presenting a CAFs tumor microenvironment with abundant tumor promoting stromal cells, CD204 (+) TAMs, and podoplanin (+) CAFs, which contributes to an increase in aggressive behavior in lung adenocarcinoma with hypoxic regions [17]. Hypoxia also induces overexpression of chemokine (C–C motif) ligand 28 (CCL28), which enhances angiogenesis and metastasis of lung adenocarcinoma [18]. However, further studies on the relationship between LUAD and hypoxia are still lacking. Considering that hypoxia in LUAD is regulated by a variety of complex genes, in this study, to evaluate the role of hypoxia in LUAD, we identified hypoxia-related genes (HRGs) by weighted gene co-expression network analysis (WGCNA) and identified HRGs related to the prognosis of LUAD patients by least absolute shrinkage and selection operator (LASSO) regression to form hypoxia-related prognostic risk score (HPRS) model to predict the prognosis of LUAD. Finally, this study aims to explore the biological effects mediated by HPRS and provide a new perspective for cancer treatment.

## 2. Materials and Methods

### 2.1. LUAD Datasets and Genes Participating in Hypoxia

The RNA-sequencing spectrum and matching clinical information of LUAD were downloaded from The Cancer Genome Atlas (TCGA), and 500 primary tumor samples were included. The RNA-seq data of LUAD in GEO were retrieved by logging into the Gene Expression Omnibus (GEO, https://www.ncbi.nlm.nih.gov/geo/) portal, and the RNA-seq data and corresponding clinical features of GSE31210 and GSE50081 datasets were downloaded, respectively. 226 cases of LUAD samples were included in GSE31210, and 398 samples were included in GSE50081. 200 hypoxia-related genes (Supplementary Table 1) were searched from Molecular Signatures Database (MsigDB, http://www.gsea-msigdb.org/gsea/index.jsp).

### 2.2. Analysis for Clinical Features of Hypoxia

Based on the expression profile of the hypoxia gene, single-sample gene set enrichment analysis (ssGSEA) was performed by gene set variation analysis (GSVA) package in the TCGA dataset to quantified hypoxia in LUAD samples. Z-score scaling was used for the ssGSEA score. Univariate and multivariate Cox regression analyses were performed to evaluate the correlation between hypoxia and clinical features and the LUAD prognosis. The samples were divided into different subgroups according to each clinical feature provided in TCGA, and the hypoxia ssGSEA score of each subgroup was analyzed under each clinical feature.

### 2.3. Weighted Gene Co-Expression Network Analysis

The WGCNA was constructed using the genes in the TCGA and helps calculate the weighted adjacency matrix by using the power (*β*) value as a soft threshold. Among all the soft thresholds, the *β* value with the highest average connectivity was selected according to the scale-free topology criterion >0.85. According to the cutreeDynamic function, the genes with similar expression patterns were clustered into the same module by hierarchical clustering tree. The minimum size of each module was set to 30, and the best cut-off height of 0.25 was selected to combine similar modules. Next, the module significance (MS) was calculated to evaluate the module eigengene in relationship with hypoxia.

### 2.4. Establishment and Validation of Hypoxia-Related Prognostic Risk Score Model

For the module with the highest correlation with hypoxia, the genes were extracted for univariate Cox regression analysis. LASSO is a common algorithm for eliminating collinearity of variables in the construction of prognostic models. The genes obtained from univariate Cox regression analysis with *p* < 0.05 were used in LASSO penalized Cox regression analysis, which was performed by R package “glmnet.” Stepwise regression can select the genes that are minimized by Akaike Information Criterion (AIC) to obtain the best model fitting based on the genes retained by LASSO regression [19]. Then, the stepAIC method in MASS package was used to identify the prognostic factors. Based on Cox regression coefficient and gene expression level, HPRS model was established, and the formula is as follows: HPRS score = ∑*β*i × Expi. In each dataset, by setting the normalized HPRS based on *z*-score = 0 as the grouping standard, the samples are divided into high HPRS group and low HPRS group. Time-dependent receiver operating characteristic curve (ROC) and survival analysis were carried out on the HPRS model.

### 2.5. Exploration of Function and Tumor Immunity

The annotated gene sets from Hallmark database were downloaded and analyzed for functional differences between low and high HPRS using gene set enrichment analysis (GSEA). ssGSEA was performed by R packet “GSVA” to explore the pathways related to HPRS. The immune and matrix scores of low HPRS and high HPRS were calculated by ESTIMATE. CIBERSORT was used to evaluate the abundance of 22 kinds of immune cells in each HPRS group.

### 2.6. Evaluation of Predictive Value of HPRS in Immunotherapy and Chemotherapy Response

To evaluate the predictive value of HPRS in immunotherapy response, the expression data of immune checkpoints were collected from HisgAtlas database, and the expression of immune checkpoints in high HPRS and low HPRS groups was analyzed. T cell dysfunction and exclusion (TIDE; https://tide.dfci.harvard.edu/) algorithm was calculated to characterize tumor immune evasion mechanism [20]. pRRophetic package estimated the half-maximal inhibitory concentration (IC50) of candidate chemotherapeutic drugs to determine the correlation between different HPRS groups and chemotherapeutic drugs.

### 2.7. Development of Decision Tree and Nomogram

We introduce all the basic clinical characteristics (age, gender, T stage, N stage, M stage, and AJCC stage) and HPRS of LUAD samples in TCGA into R package “rpart” to create an automatic decision tree to divide patients into different subgroups. A nomogram was constructed with significant clinical features screened by univariate Cox regression and HPRS by the RMS package. The performance of nomogram in predicting the prognosis of LUAD was evaluated by time-dependent ROC, calibration curve, and decision curve analysis (DCA).

### 2.8. Statistical Analysis

Statistical analysis was performed by R software (version 4.0.2, https://www.R-project.org). Univariate and multivariate Cox regression analysis was utilized to calculate the hazard ratios. Somatic mutation data were analyzed using maftools package. Kaplan–Meier survival curve was visualized by the “survminer” R package. The ROC curve was generated using the “timeROC” R package. The correlations were determined using Pearson's correlation analysis. Wilcoxon test was used for comparison of two groups. Kruskal–Wallis test was used for comparison of multiple groups. All statistical *p* values were two-sided, which <0.05 as statistical difference.

## 3. Results

### 3.1. Relationship between Hypoxia Score and Pathological Variables of LUAD

**Figure 1:**
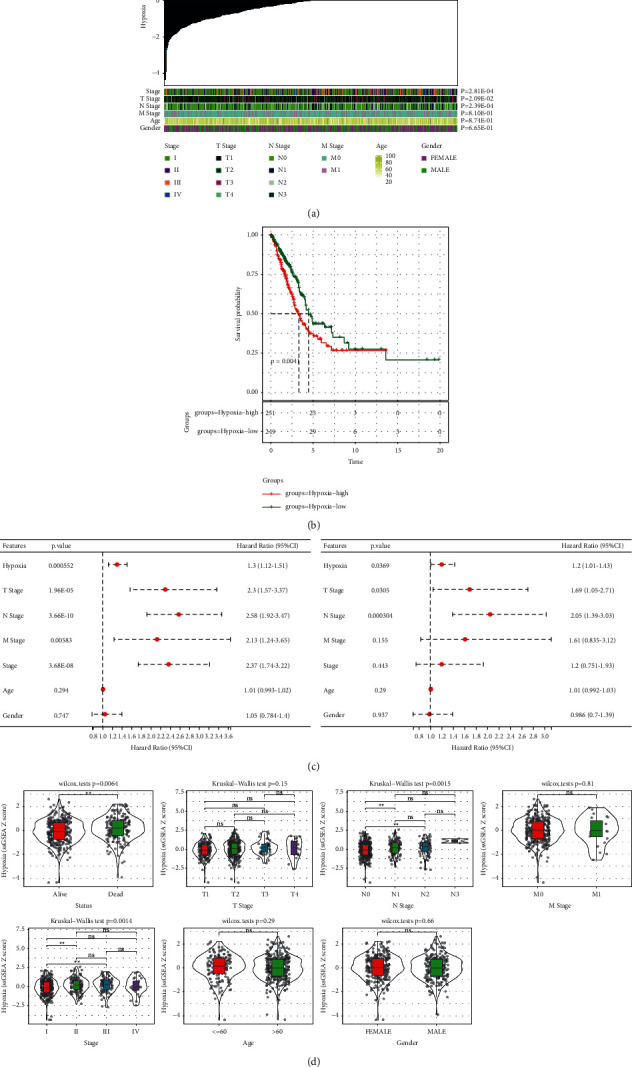
(a): LUAD clinicopathological variables under different hypoxia scores. (b): The Kaplan–Meier survival curve of distinct hypoxia score group. (c): Univariate (left) and multivariate Cox regression analysis (right) of hypoxia and LUAD clinical variables. (d): The evaluation of hypoxia score was grouped according to different clinical variables (age, gender, status, T stage, N stage, M stage, and AJCC stage) of LUAD.

We used hypoxia score as an index to evaluate the hypoxia characteristics of cancer cells. First, the hypoxia score of each LUAD patient in the TCGA was calculated by ssGSEA, and all the normalized hypoxia scores were arranged in increasing order. AJCC stage, T stage, N stage, M stage, age, and gender were evaluated under different hypoxia scores. Different hypoxia scores showed significantly different AJCC stages, T stages, and N stages. Higher hypoxia score means later AJCC stage, T stage, and N stage (Figure 1(a)). And the 10-year survival rate of patients with high hypoxia score was significantly lower than that of patients with low hypoxia score (Figure 1(b)). Univariate and multivariate Cox regression analysis indicated that hypoxia and T stage had significant prognostic value (Figure 1(c)). Patients with LUAD were divided into groups by age, gender, status, T stage, N stage, M stage, and AJCC stage, respectively. Analysis of hypoxia score in different clinical variables showed that there was no significant difference in hypoxia score in different ages, genders, T stages, and M stages. The hypoxia score of survival patients was significantly lower than that of death patients. Among the four N stages, the later the N stage, the higher the hypoxia score of patients. Moreover, hypoxia score was significantly positively correlated with AJCC stage (Figure 1(d)).

### 3.2. Identification of Hypoxia-Related Modules

**Figure 2:**
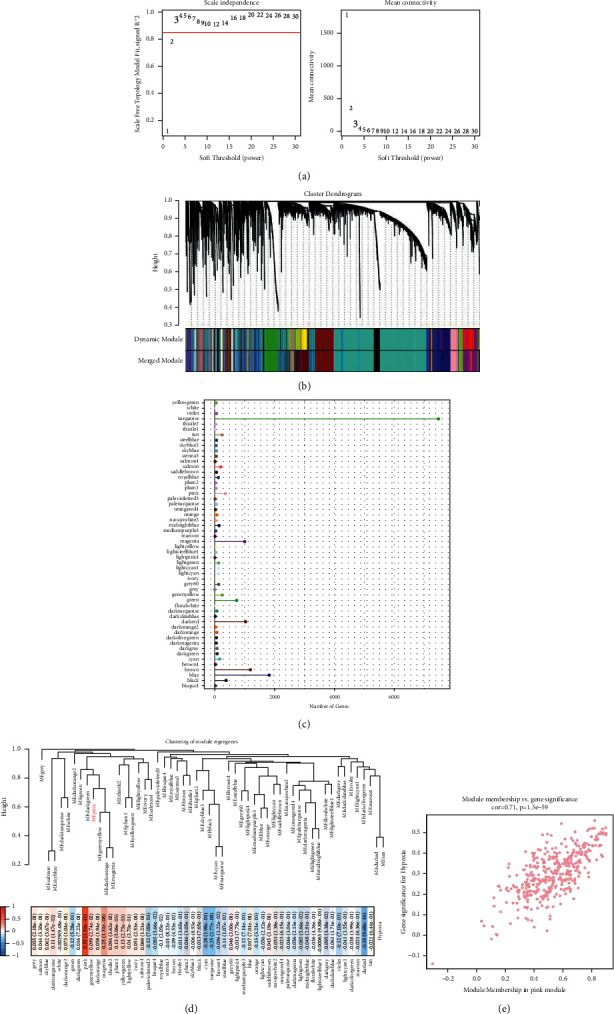
Identification of hypoxia-related modules. (a): Scale-free fitting index and average connectivity of different soft thresholds. (b): Gene dendrogram generated by hierarchical clustering. (c): The number of co-expressed genes contained in each module. (d): Relationships between module eigengenes and hypoxia; red represents positive correlation, and blue represents negative correlation. (e): The correlation between module membership (MM) and gene significance (GS) of pink module.

The WGCNA algorithm was used to construct the weighted gene co-expression module of LUAD. When the soft threshold reaches 16, the scale-free topology fitting index is greater than 0.85 (Figure 2(a)). Through clustering, all genes were assigned into 54 gene modules (Figure 2(b)). Each module contains a different number of co-expressed genes. Among them, turquoise and blue modules have the largest number of genes among all modules (Figure 2(c)). To study the correlation between hypoxia and module eigengenes (ME) and the connectivity of ME, the module-character relationship was evaluated. Pink module and magenta module showed close positive correlation with hypoxia. There was a strong negative correlation between dark and cyan module and hypoxia (Figure 2(d)). Based on the correlation coefficient greater than 0.5, this study takes the genes in the pink module as the research object. The correlation between module membership (MM) and gene significance (GS) of pink module is shown in Figure 2(e).

### 3.3. Construction and Verification of HPRS Model Based on Genes in Pink Module

**Figure 3:**
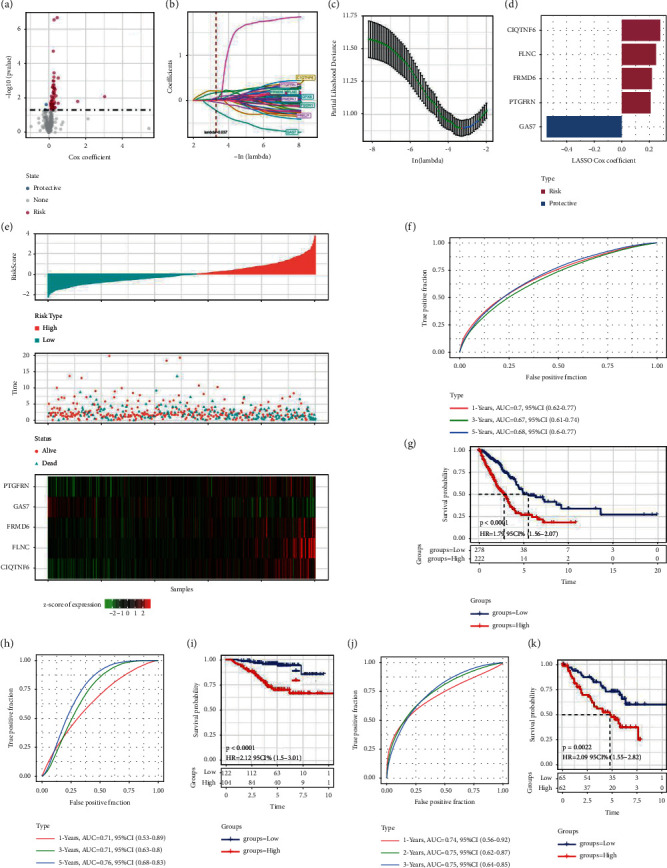
Establishment and validation of HPRS for predicting the prognosis of LUAD. (a): Univariate Cox regression analysis of genes in pink module and LUAD survival. (b): The cross-validation for penalty parameter *λ* selection in the LASSO regression. (c): Partial likelihood deviance for the LASSO coefficient profile. (d): The LASSO Cox coefficients of 5 hypoxia-related genes. (e): The relationship between the arrangement of HPRS of each LUAD patient in TCGA and patient survival and the expression of 5 genes. (f): ROC curve and AUC of 1-, 3-, and 5-year survival in TCGA. (g): The survival curve of LUAD patients with high HPRS and patients with low HPRS in TCGA. (h): The AUC for 1-year, 3-year, and 5-year ROC in external validation set. (i): Survival analysis of patients with different HPRS in external validation set GSE31210. (j): The sensitivity and specificity of ROC curve in predicting 1-, 3-, and 5-year survival in GSE50081. (k): Kaplan–Meier curve of patients with different HPRS in GSE50081.

Univariate Cox regression analysis of genes in pink module and LUAD survival identified 64 genes significantly related to LUAD survival (Figure 3(a)). LASSO and multivariate Cox regression analysis confirmed that C1QTNF6, FLNC, FRMD6, PTGFRN, and GAS7 were used to create the HPRS model (Figures 3(b) and 3(c)). The LASSO Cox coefficient of the first four is above 0, which means that the expression of these four genes is associated with poor LUAD prognosis. The LASSO Cox coefficient of GAS7 is less than 0, suggesting that its expression is related to a better prognosis (Figure 3(d)). The HPRS of each LUAD patient in TCGA was calculated and sorted using HPRS model. The increase of HPRS was associated with a decrease in time of death and an increase in mortality in patients with LUAD. The expression of genes with LASSO Cox coefficient greater than 0 was upregulated with the increase of HPRS, and the expression of genes with LASSO coefficient less than 0 was reversed (Figure 3(e)). For patients with high HPRS in the TCGA-LUAD dataset, survival was significantly lower than for patients with low HPRS (Figure 3(g)). In this dataset, the AUC of 1-year, 3-year, and 5-year ROC was 0.7, 0.67, and 0.68, respectively (Figure 3(f)). In the external validation sets GSE31210 and GSE50081, patients with low HPRS were more likely to die earlier and have a poor prognosis than patients with high HPRS (Figures 3(i) and 3(k)). For two independent external verification sets, the AUC of 1-year, 3-year, and 5-year ROC was very high, not only exceeding 0.7 for all, but also the AUC of OS was more than 0.75 in 5 years (Figures 3(h) and 3(j)), indicating that HPRS has acceptable accuracy in all three cohorts studied.

### 3.4. The Relation between Genetic Alterations and HPRS in LUAD

**Figure 4:**
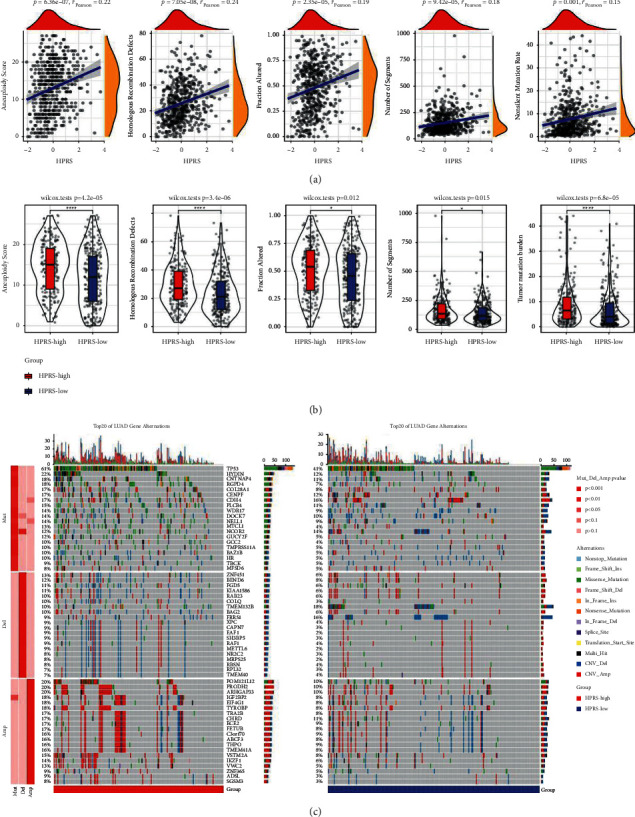
The relation between genetic alterations and HPRS in LUAD. (a): The Pearson correlation between HPRS and aneuploidy score, homologous recombination defects, fraction altered, number of segments, and tumor mutation burden, respectively. (b): Differences in aneuploidy score, homologous recombination defects, fraction altered, number of segments, and tumor mutation burden between the two HPRS groups. (c): Comparison of mutation spectrum between high and low HPRS groups. ^*∗*^*p* < 0.05, ^*∗∗∗∗*^*p* < 0.0001.

To determine whether genetic alterations affect HPRS, the correlation between HPRS and genomic stability-related indexes (aneuploidy score, homologous recombination defects, fraction altered, number of segments, and tumor mutation burden) was analyzed. All five indicators showed a strong positive correlation with HPRS, respectively (Figure 4(a)). The differences of aneuploidy score, homologous recombination defects, fraction altered, and number of segments and tumor mutation burden between the two HPRS groups were compared. The results showed that there were significant differences in these indexes between high and low HPRS patients, and the aneuploidy score, homologous recombination defects, fraction altered, and number of segments and tumor mutation burden of high HPRS patients were significantly higher than those of low HPRS patients (Figure 4(b)). From the results of mutation spectrum analysis, we found that the two HPRS groups had a wide range of variation types, and the main types were missense mutation and nonsense mutation. Among all the mutated genes, the mutation rates of TP53 and HYDIN in both HPRS groups were quite high, and the mutation rate in patients with high HPRS was much higher than that in patients with low HPRS. The copy number variation (CNV) analysis of the two HPRS groups showed that the CNV amplification in the high HPRS group was generally higher than that in the low HPRS group. The CNV deletion of TMEM132 B and FRRS1 in low HPRS group was significantly higher than that in high HPRS group (Figure 4(c)). This indicated that genetic alterations are highly heterogeneous between high and low HPRS groups.

### 3.5. The Role of HPRS in Signal Pathway Regulation and TME Cell Infiltration

**Figure 5:**
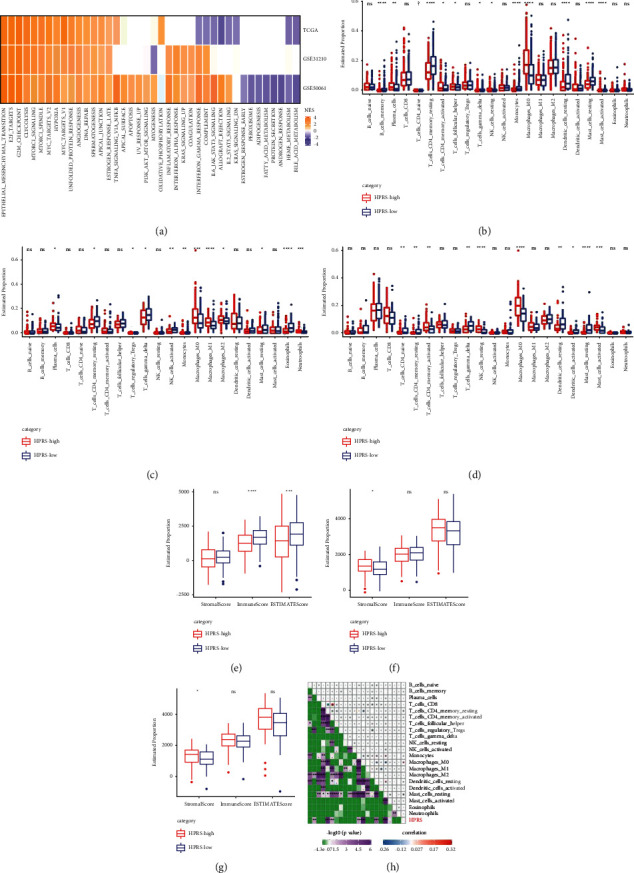
The role of HPRS in signal pathway regulation and TME cell infiltration. (a): High HPRS versus low HPRS pathway activity in each cohort. (b): The proportion of 22 tumor infiltrating cells between high and low HPRS groups in TCGA-derived LUAD patients. (c, d): The proportion of 22 tumor infiltrating cells between high and low HPRS groups in GSE31210 and GSE50081. (e): Stromal score and immune score differences between high and low HPRS in TCGA-derived LUAD patients. (f, g): Stromal score and immune score differences between high and low HPRS in GSE31210 and GSE50081. (h): Analysis of the correlation between HPRS and the proportion of 22 kinds of immune cells in TCGA-derived LUAD patients. ^*∗*^*p* < 0.05, ^*∗∗*^*p* < 0.01, ^*∗∗∗*^*p* < 0.001, ^*∗∗∗∗*^*p* < 0.0001.

To study the significance of HPRS in tumor development, the pathway activity of high HPRS versus low HPRS in each cohort was analyzed. In patients with TCGA-derived LUAD, there were 17 significantly activated signaling pathways and 8 significantly suppressed pathways in high HPRS compared with low HPRS. In three cohorts, 15 pathways were activated in patients with high HPRS, among which epithelial-mesenchymal transition, E2F targets, G2M checkpoint, MYC targets, angiogenesis, and DNA repair were all important pathways affecting the occurrence and development of cancer (Figure 5(a)). Then, we selected the gene expression profile corresponding to LUAD samples in TCGA cohort, performed ssGSEA analysis using R software package GSVA, calculated the scores of each sample on different KEGG functions, and computed the correlation between the scores of these functions and HPRS. HPRS was positively correlated with mismatch repair, DNA replication, homologous recombination, cell cycle, nucleotide excision repair, and other pathways (Supplementary Figure 1(a)). In addition, Pearson correlation analysis also showed that HIF-1 *α* and hypoxia score were significantly positively correlated with HPRS, respectively (Supplementary Figures 1(b) and 1(c)). The role of HPRS in TME cell infiltration was then evaluated in three cohorts. By comparing the proportion of 22 tumor infiltrating cells in high and low HPRS groups, it was found that there was a significant difference in the proportion of 12 tumor immune infiltrating cells in TCGA-LUAD between high and low HPRS groups (Figure 5(b)). 12 and 14 tumor infiltrating immune cells were found in GSE31210 and GSE50081, respectively, showing significant differences between high and low HPRS groups (Figures 5(c) and 5(d)). Then, the TME-related stromal score and immune score between high and low HPRS were analyzed by ESTIMATE. In TCGA-derived LUAD patients, there was no significant difference in stromal score between high and low HPRS, but immune score in high HPRS group was significantly lower than that in low HPRS group (Figure 5(e)). In the other two LUAD cohorts, the stromal score of the high HPRS group was significantly higher than that of the low HPRS group, and there was no statistical difference in immune score between the two groups (Figures 5(f) and 5(g)). The correlation analysis between HPRS and 22 immune cells showed that HPRS was significantly correlated with the proportion of memory B cells, resting memory CD4 T cells, activated memory CD4 T cells, monocytes, M0 macrophages, and resting dendritic cells and resting mast cells (Figure 5(h)). These results suggest that HPRS is a potential prediction tool for evaluating TME in LUAD.

### 3.6. The Role of HPRS in Predicting the Immunotherapy Response and Chemotherapy Effect of LUAD

**Figure 6:**
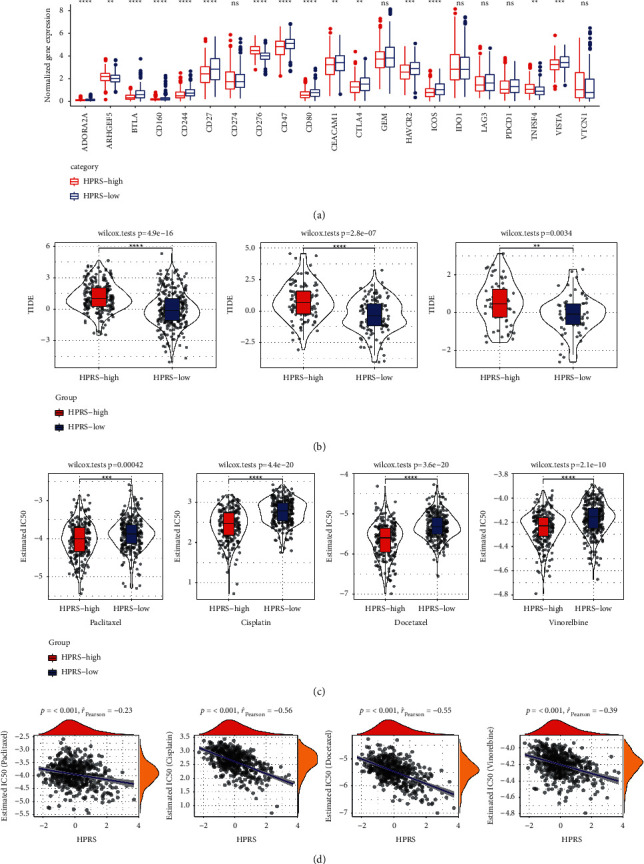
The role of HPRS in predicting the immunotherapy response and chemotherapy effect of LUAD. (a): The normalized expression (Log2 (FPKM + 1)) of immune checkpoints in high HPRS and low HPRS groups. (b): In TCGA (left), GSE31210 (center), and GSE50081 (right), the difference of TIDE score between high HPRS group and low HPRS group. (c): Estimated IC50 of paclitaxel, cisplatin, docetaxel, and vinorelbine in different HPRS groups. (d): Correlation between HPRS and estimated IC50 of paclitaxel, cisplatin, docetaxel, and vinorelbine. ^*∗*^*p* < 0.05, ^*∗∗*^*p* < 0.01, ^*∗∗∗*^*p* < 0.001, ^*∗∗∗∗*^*p* < 0.0001.

We explored the role of HPRS in predicting the immunotherapy response and chemotherapy effect of LUAD. First, the expression of immune checkpoints in high HPRS group and low HPRS group was examined, and we found that there was a significant difference in the expression of most immune checkpoints between the two groups (Figure 6(a)). In addition, the potential responses to immunotherapy in the high HPRS and low HPRS groups were compared from the point of view of evaluating TIDE scores. We found that in all the three LUAD cohorts studied, the TIDE score of the high HPRS group was always significantly higher than that of the low HPRS group (Figure 6(b)). Furthermore, the responses of different HPRS groups to chemotherapeutic drugs paclitaxel, cisplatin, docetaxel, and vinorelbine were investigated in patients with LUAD derived from TCGA. Comparing the estimated IC50 of each chemotherapeutic drug in different HPRS groups, the results showed that the patients with high HPRS had significantly higher sensitivity to paclitaxel, cisplatin, docetaxel, and vinorelbine than the patients with low HPRS (Figure 6(c)). And the Pearson correlation analysis between HPRS and IC50 of four chemotherapeutic drugs showed that HPRS was significantly negatively correlated with IC50 values of paclitaxel, cisplatin, docetaxel, and vinorelbine (Figure 6(d)).

### 3.7. Comparison of Prognostic Ability between HPRS and Traditional Clinical Indexes

**Figure 7:**
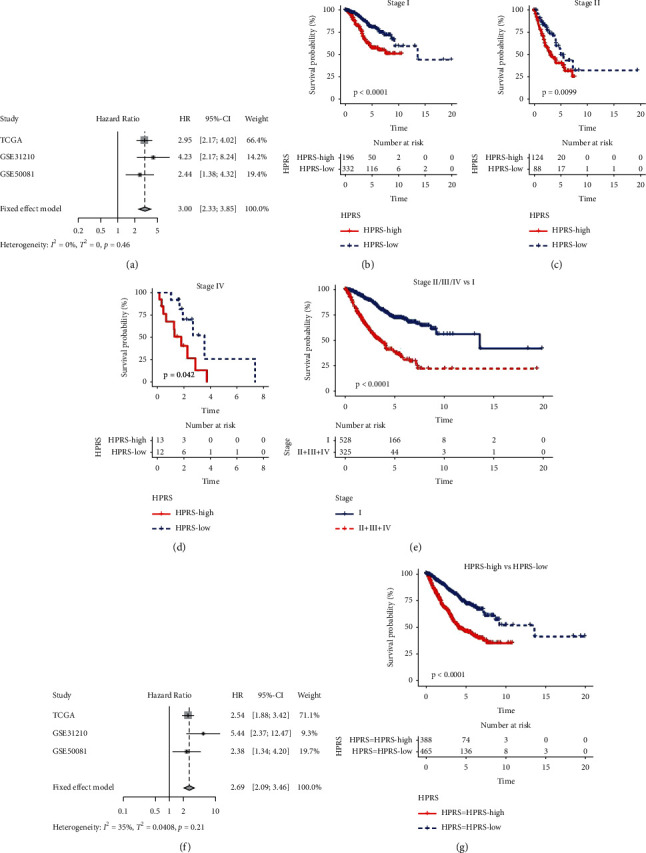
Comparison of prognostic ability between HPRS and traditional clinical indexes. (a): Meta-analysis was performed to calculate the pooled HR of TNM staging classification. (b-d): OS of patients undergoing HPRS grouping in stage I, stage II, and stage IV patients, respectively. (e): OS analysis of stage II-IV patients compared with stage. (f): Meta-analysis of the correlation between HPRS and LUAD prognoses in 853 LUAD samples. (g): The survival curves of high HPRS group and low HPRS group were measured in 853 LUAD samples.

LUAD patient data from TCGA and independent cohorts GSE31210 and GSE50081 were combined, and a total of 853 LUAD samples were included. The meta-analysis confirmed that LUAD patients with high TNM stage have poor prognosis (Figure 7(a)). We also found that there was a significant difference in OS among patients with stage I, stage II, and stage IV (Figures 7(b)–7(d)). Compared with stage I, the OS of patients with stage II-IV was significantly shorter (Figure 7(e)). Additionally, meta-analysis was utilized to estimate the relationship between HPRS and the prognosis of LUAD. The results presented in Figure 7(f) showed that the comprehensive HR was 2.69, indicating that there was a correlation between HPRS and the prognosis of LUAD, and there was no significant heterogeneity (Figure 7(f)). Subsequent survival analysis showed that patients with high HPRS had significantly longer OS than patients with low HPRS (Figure 7(g)). Therefore, HPRS is also a prognostic factor for poor prognosis of LUAD.

### 3.8. Risk Stratification of LUAD Patients by Decision Tree and Nomogram

**Figure 8:**
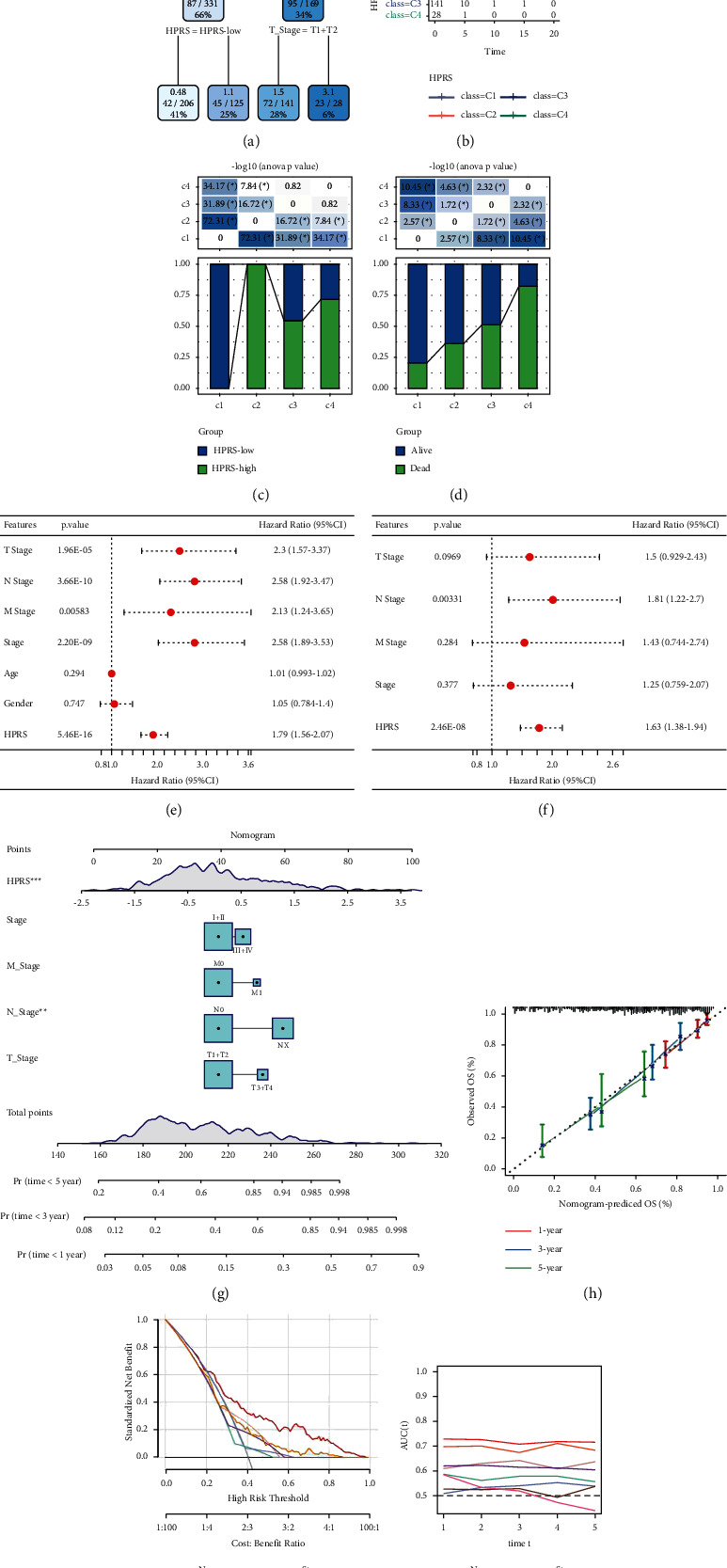
Construction of decision tree and nomogram for risk stratification of LUAD patients. (a): The decision tree based on LUAD clinicopathological variables (age, gender, T stage, N stage, M stage, and AJCC stage) in TCGA and HPRS. (b): The Kaplan–Meier curve of OS in four different risk subgroups. (c): HPRS distribution in each risk subgroup. (d): The proportion of survival and mortality included in the risk subgroup. (e, f): Univariate Cox regression and multivariate Cox regression of clinicopathological variables and HPRS. (g): A nomogram is constructed by combining the important clinical parameters obtained by univariate Cox regression and HPRS using multivariate Cox regression. (h): The calibration curve shows the accuracy of nomogram in predicting 1-, 3-, and 5-year OS in patients with LUAD. (i): The decision curve for important clinical parameters obtained from univariate Cox regression, HPRS, and nomogram. (j): The tROC curve of all the clinicopathological variables given in TCGA and HPRS and nomogram.

To better stratify the risk of LUAD patients, the clinicopathological variables of LUAD in TCGA (age, gender, T stage, N stage, M stage, and AJCC stage) and HPRS were used to construct a decision tree. The whole decision tree consisted of two decision nodes and four terminal nodes, so patients were divided into four different risk subgroups (C1, C2, C3, and C4) (Figure 8(a)). These risk subgroups showed significantly different survival outcomes (Figure 8(b)). All cases in C1 were of low HPRS, all cases in C2 were of high HPRS, nearly 55% of patients were with high HPRS, nearly 45% of patients were with low HPRS in C3, and the proportion of patients with high HPRS in C4 was much larger than the proportion of patients with low HPRS (Figure 8(c)). From C1 to C4, the proportion of deaths increased gradually (Figure 8(d)). Univariate Cox regression and multivariate Cox regression analysis for clinicopathological variables and HPRS showed that N stage or HPRS may be an independent prognostic factor for poor prognosis of LUAD (Figures 8(e) and 8(f)).

To optimize the risk assessment of LUAD patients, a nomogram was constructed by combining the important clinical parameters obtained by univariate Cox regression and HPRS using multivariate Cox regression (Figure 8(g)). The calibration curve showed that the 1-year, 3-year, and 5-year prediction lines of nomogram were close to the 45-degree dotted line, indicating that the line chart had high precision (Figure 8(h)). Decision curve showed that nomogram generated the highest returns (Figure 8(i)). Of all the clinicopathological variables given in TCGA-LUAD, AUC of tROC of nomogram had the highest accuracy in finding nomogram, indicating that it is the most effective in predicting LUAD survival (Figure 8(j)).

## 4. Discussion

Hypoxia is a landmark pathological feature in the development of LUAD. TME is shaped by the changes of genome, transcriptome, and proteome to increase the malignant potential [21]. Common detection methods of hypoxia include the use of immunohistochemistry to identify hypoxia-induced proteins and imaging techniques to visualize hypoxia sensitivities. In the past few years, histological techniques and bioinformatics analysis tools have greatly expanded the study of cancer hypoxia, and a new method of incorporating hypoxia gene signatures has emerged [22]. Compared with 21-gene hypoxia signature reported by Zhang, et al., which has been proved to have the ability to indicate hypoxia in hepatocellular carcinoma [23]. The hypoxia-immune-based gene signature developed by Yang et al. can reflect the hypoxia state in TME of triple negative breast cancer [23]. In this study, we also explored the hypoxia characteristics of LUAD and constructed HPRS to evaluate the relationship between hypoxia and genetic alterations, TME, and immunotherapy.

Based on the 200 hypoxia-related genes, we performed ssGSEA to obtain the hypoxia score of each LUAD patient as an indicator of cancer cell hypoxia. Here, higher hypoxia score indicated later AJCC stage, T stage, N stage, shorter OS, and hypoxia score was positively correlated with patient survival status, N stage, and AJCC stage. It means that hypoxia score may positively promote the progress of LUAD. To further understand the role of hypoxia in LUAD, we passed WGCNA and LASSO cox methods and selected the five genes most related to hypoxia, which ensured the exclusiveness of the HPRS we established in patients with LUAD.

In fact, the importance of five genes in HPRS in cancer has been reported in several previous studies. C1q/tumor necrosis factor-related protein 6 (C1QTNF6) is a glycoprotein composed of 259 amino acids, which plays an important role in predicting the prognosis of many cancers, including lung adenocarcinoma, bladder cancer, and gastric cancer [24–26]. A previous study revealed that the increased expression of filamin C (FLNC) in hepatocytes is associated with microvascular invasion and poor prognosis [27]. Microvascular invasion is a kind of TME change induced by hypoxia [28]. Therefore, it also indirectly indicates that there is a correlation between FLNC and hypoxia. Cell test and tissue test showed that FERM domain containing 6 (FRMD6) had tumor inhibitory effect in prostate cancer [29] and thyroid cancer [30]. In contrast, FRMD6 is a wind risk factor that threatens the prognosis of LUAD in our study. Prostaglandin F2 receptor negative (PTGFRN) regulator is overexpressed in glioblastoma multiforme and associated with poor survival, downregulates its expression of radiosensitized cancer cells, and effectively slows down the rate of cell proliferation and tumor growth [31]. In a study related to nonsmall-cell lung cancer, it has been reported that the high level of mRNA of growth arrest-specific 7 (GAS7) is related to the improvement of overall survival, and downregulation of its expression can antagonize the resensitivity of gefitinib in lung cancer cells [32]. In this study, HPRS could accurately predict the OS of patients with LUAD, and five key genes in HPRS are also supported as potential targets of LUAD.

The evaluation of mutated genes that drive tumors is a milestone in cancer detection and treatment selection. We tried to describe the impact of HPRS on genetic alterations. The indicators related to genomic stability, including aneuploidy score, homologous recombination defects, fraction altered, and number of segments and tumor mutation burden, showed a strong positive correlation with HPRS. We also revealed the variation types of different HPRS groups. Missense mutation and nonsense mutation were the main variation types in LUAD. As the most common single gene mutation in human cancer, TP53 has a high mutation rate in patients with high HPRS. The overall frequency of somatic mutation and CNV in patients with high HPRS was also much higher than that in patients with low HPRS.

TME is a complex network of immune cells, endothelial cells, fibroblasts, and various signal molecules [33]. Hypoxia is one of the most important factors in shaping TME [34]. In our research work, we explored the role of HPRS in TME in patients with LUAD. The TME of different HPRS groups was described from the point of view of evaluating tumor infiltrating immune cells, matrix components, and immune components. At the same time, more and more literature shows that hypoxia microenvironment can reduce the sensitivity of ICI treatment [34]. Our study indicated that patients with high HPRS are more likely to have immune escape, which means that they are not sensitive to immunotherapy. Finally, the clinical decision tree integrating the clinicopathological variables of HPRS and LUAD and nomogram optimized the risk stratification of LUAD patients.

To sum up, in combination with WGCNA and LASSO regression analysis, this study created a HPRS that helps to characterize genetic alterations and TME in LUAD, predicting the adaptation of LUAD to immunotherapy and chemotherapy drugs. Moreover, HPRS can be used as a potential predictive tool to predict the OS of LUAD patients and guide personalized clinical practice.

## Data Availability

The datasets analyzed in this study could be found in [GSE31210] at [https://www.ncbi.nlm.nih.gov/geo/query/acc.cgi?acc=GSE31210] and in [GSE50081] at [https://www.ncbi.nlm.nih.gov/geo/query/acc.cgi?acc= GSE50081].

## Supplementary Materials

Supplementary Materials Supplementary Table 1 The name of 200 hypoxia genes. Supplementary Figure 1 Pearson correlation analysis between HPRS and KEGG pathway and HIF-1 *α*. a: Heatmap demonstrating normalized enrichment scores (NESs) of Hallmark pathways calculated by comparing HPRS high with HPRS low. b: Pearson correlation analysis between HPRS and HIF-1 *α*. c: Pearson correlation analysis between HPRS and hypoxia score.
