# Supplementary material for: A New Hypoxia-Related Prognostic Risk Score (HPRS) Model Was Developed to Indicate Prognosis and Response to Immunotherapy for Lung Adenocarcinoma
Source: J Oncol. 2022 Jul 30;2022:6373226. doi: 10.1155/2022/6373226 (PMC9356881; doi:10.1155/2022/6373226)
Supplement: Supplementary Materials — Supplementary Table 1 The name of 200 hypoxia genes. Supplementary Figure 1 Pearson correlation analysis between HPRS and KEGG pathway and HIF-1 α. a: Heatmap demonstrating normalized enrichment scores (NESs) of Hallmark pathways calculated by comparing HPRS high with HPRS low. b: Pearson correlation analysis between HPRS and HIF-1 α. c: Pearson correlation analysis between HPRS and hypoxia score. [file 6373226.f1.zip › 6373226.f1/Supplementary Table 1.pdf]

200 hypoxia genes
ACKR3
ADM
ADORA2B
AK4
AKAP12
ALDOA
ALDOB
ALDOC
AMPD3
ANGPTL4
ANKZF1
ANXA2
ATF3
ATP7A
B3GALT6
B4GALNT2
BCAN
BCL2
BGN
BHLHE40
BNIP3L
BRS3
BTG1
CA12
CASP6
CAV1
CAVIN1
CAVIN3
CCN1
CCN2
CCN5
CCNG2
CDKN1A
CDKN1B
CDKN1C
CHST2
CHST3
CITED2
COL5A1
CP
CSRP2
CXCR4
DCN
DDIT3
DDIT4
DPYSL4
DTNA
DUSP1
EDN2
EFNA1
EFNA3
EGFR
ENO1
ENO2
ENO3
ERO1A
ERRFI1

ETS1
EXT1
F3
FAM162A
FBP1
FOS
FOSL2
FOXO3
GAA
GALK1
GAPDH
GAPDHS
GBE1
GCK
GCNT2
GLRX
GPC1
GPC3
GPC4
GPI
GRHPR
GYS1
HAS1
HDLBP
HEXA
HK1
HK2
HMOX1
HOXB9
HS3ST1
HSPA5
IDS
IER3
IGFBP1
IGFBP3
IL6
ILVBL
INHA
IRS2
ISG20
JMJD6
JUN
KDELR3
KDM3A
KIF5A
KLF6
KLF7
KLHL24
LALBA
LARGE1
LDHA
LDHC
LOX
LXN
MAFF
MAP3K1
MIF
MT1E

MT2A
MXI1
MYH9
NAGK
NCAN
NDRG1
NDST1
NDST2
NEDD4L
NFIL3
NOCT
NR3C1
P4HA1
P4HA2
PAM
PCK1
PDGFB
PDK1
PDK3
PFKFB3
PFKL
PFKP
PGAM2
PGF
PGK1
PGM1
PGM2
PHKG1
PIM1
PKLR
PKP1
PLAC8
PLAUR
PLIN2
PNRC1
PPARGC1A
PPFIA4
PPP1R15A
PPP1R3C
PRDX5
PRKCA
PYGM
RBPJ
RORA
RRAGD
S100A4
SAP30
SCARB1
SDC2
SDC3
SDC4
SELENBP1
SERPINE1
SIAH2
SLC25A1
SLC2A1
SLC2A3
SLC2A5

SLC37A4
SLC6A6
SRPX
STBD1
STC1
STC2
SULT2B1
TES
TGFB3
TGFBI
TGM2
TIPARP
TKTL1
TMEM45A
TNFAIP3
TPBG
TPD52
TPI1
TPST2
UGP2
VEGFA
VHL
VLDLR
WSB1
XPNPEP1
ZFP36
ZNF292
