# Supplementary figures and images for: A New Hypoxia-Related Prognostic Risk Score (HPRS) Model Was Developed to Indicate Prognosis and Response to Immunotherapy for Lung Adenocarcinoma
Source: J Oncol. 2022 Jul 30;2022:6373226. doi: 10.1155/2022/6373226 (PMC9356881; doi:10.1155/2022/6373226)

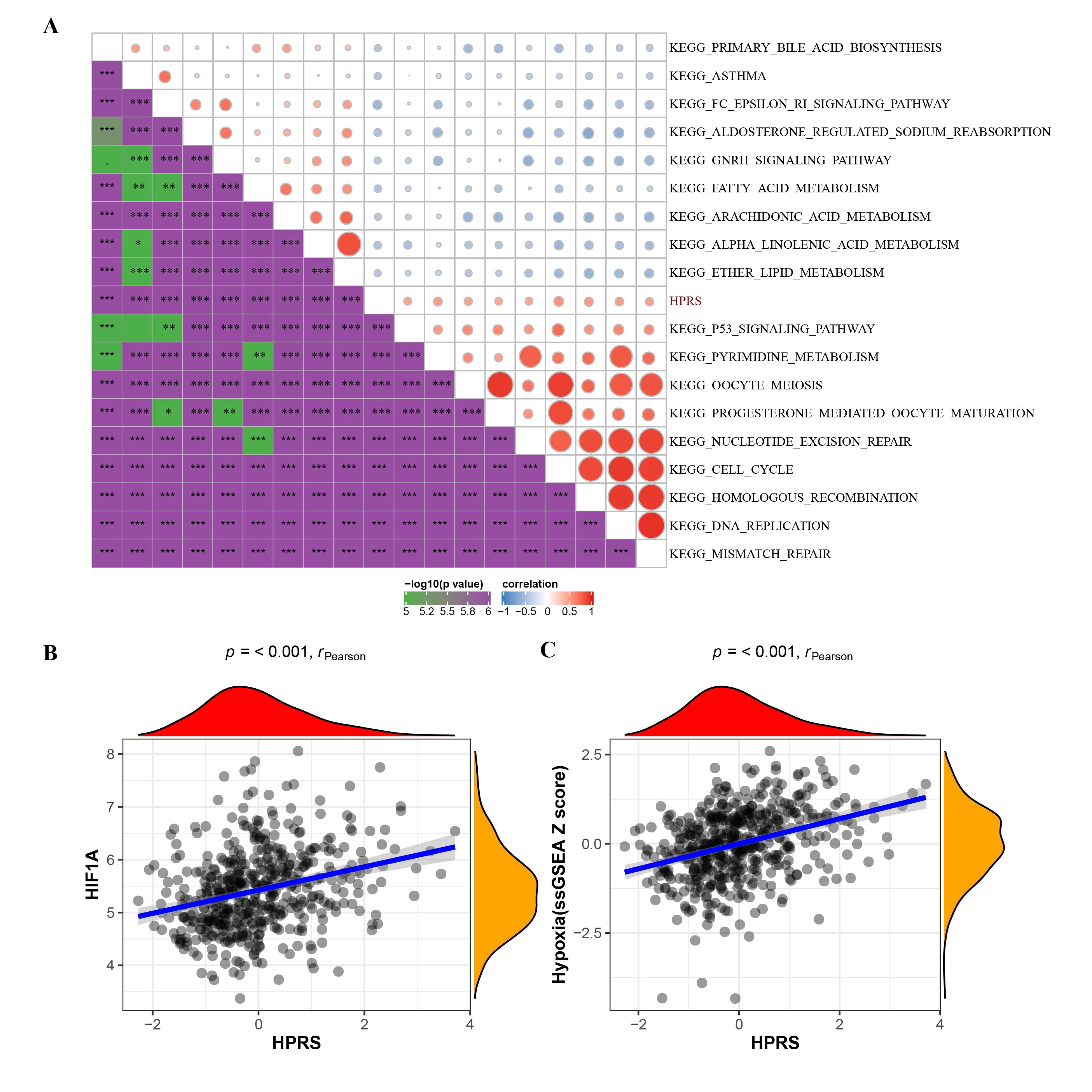

Supplement: Supplementary Materials — Supplementary Table 1 The name of 200 hypoxia genes. Supplementary Figure 1 Pearson correlation analysis between HPRS and KEGG pathway and HIF-1 α. a: Heatmap demonstrating normalized enrichment scores (NESs) of Hallmark pathways calculated by comparing HPRS high with HPRS low. b: Pearson correlation analysis between HPRS and HIF-1 α. c: Pearson correlation analysis between HPRS and hypoxia score. [file 6373226.f1.zip › 6373226.f1/Supplementary Figure 1.jpg]
